# Subtraction of Temporally Sequential Digital Mammograms: Enhancing the Detection and Classification of Malignant Masses in Breast Imaging

**DOI:** 10.1109/OJEMB.2025.3624977

**Published:** 2025-10-23

**Authors:** Kosmia Loizidou, Galateia Skouroumouni, Gabriella Savvidou, Anastasia Constantinidou, Eleni Orphanidou Vlachou, Anneza Yiallourou, Costas Pitris, Christos Nikolaou

**Affiliations:** ^1^ KIOS Research and Innovation Center of ExcellenceUniversity of Cyprus 2109 Nicosia Cyprus; ^2^ Ygia Polyclinic Limassol 3025 Limassol Cyprus; ^3^ Medical School, University of CyprusBank of Cyprus Oncology Centre 2109 Nicosia Cyprus; ^4^ EIMC Clinic Strovolos 2408 Nicosia Cyprus; ^5^ Medical School University of Cyprus, Breast Unit Nicosia General HospitalState Health Services Organization 2109 Nicosia Cyprus; ^6^ Limassol General Hospital 4131 Limassol Cyprus

**Keywords:** Breast cancer, machine learning, mammography, medical image analysis

## Abstract

*Background:* This study evaluates the performance of an automated method for detecting and classifying breast masses as Breast Imaging Reporting and Data System (BI-RADS) benign or biopsy-confirmed malignant using subtraction of temporally sequential mammograms. Mammograms from 100 women across two screening rounds (400 images: 2 views × 2 rounds × 100 cases) were retrospectively collected. The prior mammographic views were subtracted from the most recent ones, 98 image features were extracted from regions of interest, and were ranked using 8 feature selection methods. *Results:* Machine learning reduced false positives and detected masses with 97.06% accuracy and 0.92 AUC. True masses were classified as benign or malignant with 94.82% accuracy and 0.95 AUC, a significant improvement compared with state-of-the-art methods reported in the literature (0.95 vs. 0.90 AUC). *Conclusions:* The proposed approach demonstrates that temporal subtraction can improve diagnostic accuracy by up to 5%, supporting earlier detection of malignancies and enabling more personalized treatment strategies.

## I. Introduction

Breast Cancer (BC) poses a significant global health threat according to the World Health Organization [1], with the incidence rates steadily increasing, by about 0.5% annually, since 2005 [2]. Mammography, in addition to physical examination, is an effective screening modality for BC. However, despite relevant technological advancements, the diagnosis of BC remains challenging, mainly due to the ambiguous radiological appearance of breast lesions and low imaging contrast [3]. Currently, mammogram evaluation requires consensus between two expert radiologists, with a third reading if necessary to break the tie. The large population and the use of double readings contribute to increased costs and resource burden and exacerbate inefficiencies in the screening process [4].

Radiological classification of breast abnormalities as benign or suspicious relies on various parameters such as shape, texture, and temporal information [5]. Suspicious masses, characterized by irregular and spiculated boundaries, are usually larger, while benign masses exhibit rounder, well-defined boundaries without infiltrated margins [6]. However, increased breast density presents a diagnostic challenge by limiting the visibility of abnormal lesions, reducing the sensitivity of mammography by approximately 30% for women with dense breasts [7].

Over the past three decades, studies have assessed Computer-Aided Diagnosis (CAD) systems for the detection of breast masses in the current mammographic views, using feature-based machine learning. These studies reported sensitivity and specificity ranging from of 81% to 96% and 73% to 97%, respectively, for the classification of benign vs. suspicious breast masses [8], [9], [10], [11]. The main drawback of these algorithms is the significant number of False Positives (FPs) per image, which hinders their use in clinical practice. Also, they rely solely on the most recent mammographic views for the diagnosis, without performing any comparison with previous mammograms to identify recent changes. However, identifying newly developed abnormalities or rapidly changing regions between screenings is crucial for accurate diagnosis in clinical practice. Previous studies attempted to reduce FPs and recall rates by combining information from prior and recent mammographic views using temporal analysis, a technique that compares specific parts of recent and prior mammograms [9]. However, this technique does not provide any advantage when the findings are completely new, with no previous traces of the abnormality [12]. There has been no further research regarding the effect of sequential mammograms on BC diagnosis since.

All the above studies suffer from several limitations: (1) a high number of FPs per image, i.e. normal tissue incorrectly identified as suspicious masses by the algorithms, which hinders clinical adoption, (2) reliance solely on the most recent mammographic views without exploiting prior exams, which are critical for detecting interval changes, and (3) limited effectiveness in women with dense breasts, where mammographic sensitivity can be reduced by 30% [7]. No existing technology adequately addresses these fundamental limitations. This highlights the gap between current technical progress and clinically useful solutions, underscoring the need for next-generation algorithms that integrate sequential information for accurate BC diagnosis.

**Figure 1.**
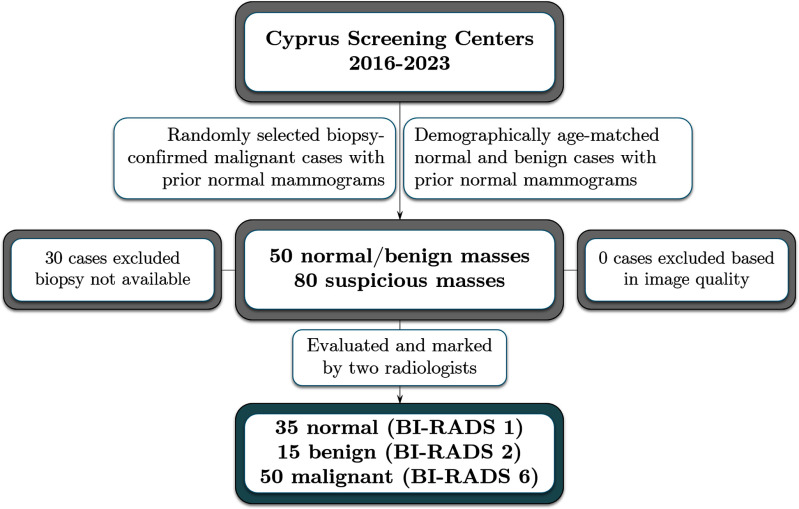
Study flowchart, showing the inclusion and exclusion criteria, and the recruitment process. *BI-RADS:* Breast Imaging Reporting and Data System.

**Figure 2.**
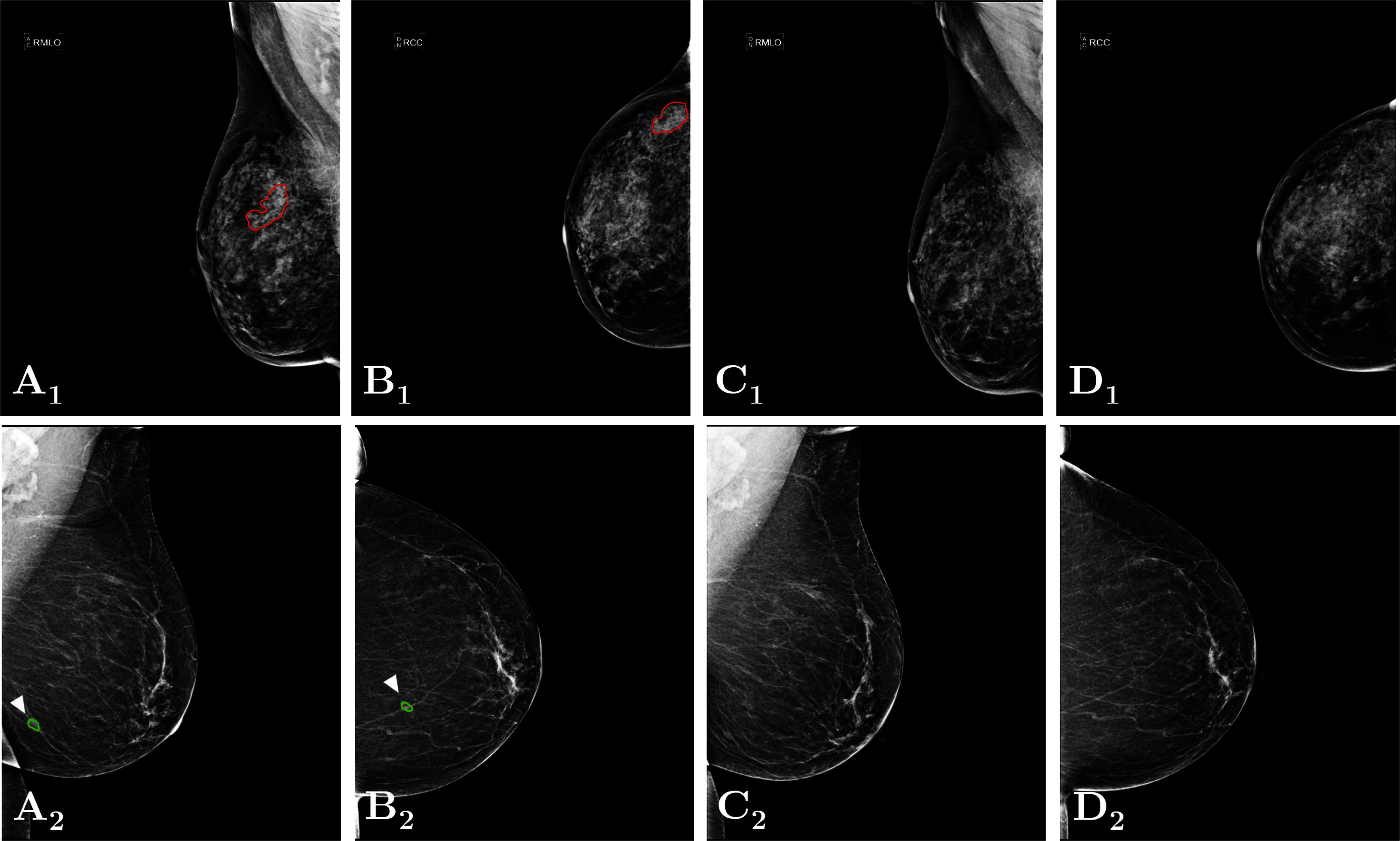
Dataset examples. (1) Mammographic views of a woman with BI-RADS breast density category *d* (Case 1, top row). (2) Mammographic views of a woman with BI-RADS breast density category *b* (Case 2, bottom row). For each case: **(A)** Most recent mammogram in Medio-Lateral Oblique (MLO) view with precise annotations of each mass. **(B)** Most recent mammogram in Cranio-Caudal (CC) view with precise annotations of each mass. **(C)** Prior MLO image. **(D)** Prior CC image. Annotations: red for biopsy-confirmed malignant mass, green for benign mass.

Temporal subtraction, introduced by this group, involves utilizing image and epidemiological features to register the entire breast area of the prior mammograms to the recent one [13], [14], [15]. This algorithm has the ability to detect newly developed abnormalities in cases where prior mammograms were entirely normal. Unlike temporal analysis, which assesses specifically lesion evolution, i.e., only if the abnormality also appears in the prior rounds, this approach utilizes mammogram subtraction to identify subtle changes between two sequential screening rounds. This enables earlier diagnosis of malignancies, improving patient outcomes, and optimizing screening strategies. This study aims to evaluate the effectiveness of the subtraction of temporally sequential mammograms, combined with advanced feature selection and machine learning techniques, for the classification of Breast Imaging Reporting and Data System (BI-RADS) benign vs. biopsy-confirmed malignant masses. A new dataset was specifically collected for this study, containing sequential mammograms from 100 patients. Details related to the inclusion and exclusion criteria, as well as the recruitment process, can be found in Fig. 1. Table 1, displays the characteristics of the selected population. Two radiologists assessed the mammograms and outlined the border of each mass, both benign and suspicious (Fig. 2).

**TABLE 1:** Characteristics of the Population and Digital Mammographic Examinations Selected for the Study

**Variable**	**Population**			
	**BI-RADS Normal**	**BI-RADS Benign**	**Malignant**	**Total**
	*(n = 35)*	*(n = 15)*	*(n = 50)*	*(n = 100)*
**Patient Age**				
*Mean $\pm$ STD*	56.5$\pm$11.4	57.8$\pm$8.1	63.6$\pm$7.2	60.2$\pm$9.6
*Median*	55	55	63.5	60.5
*Range*	40-81	48-73	48-80	40-81
*Interquartile Range*	47-63	50-65	58.7-69	53-68
**BI-RADS breast density**				
*a*	6	0	5	11
*b*	14	9	22	45
*c*	12	5	22	39
*d*	3	1	1	5

## II. Results

### A. Breast Mass Segmentation and Detection

**Figure 3.**
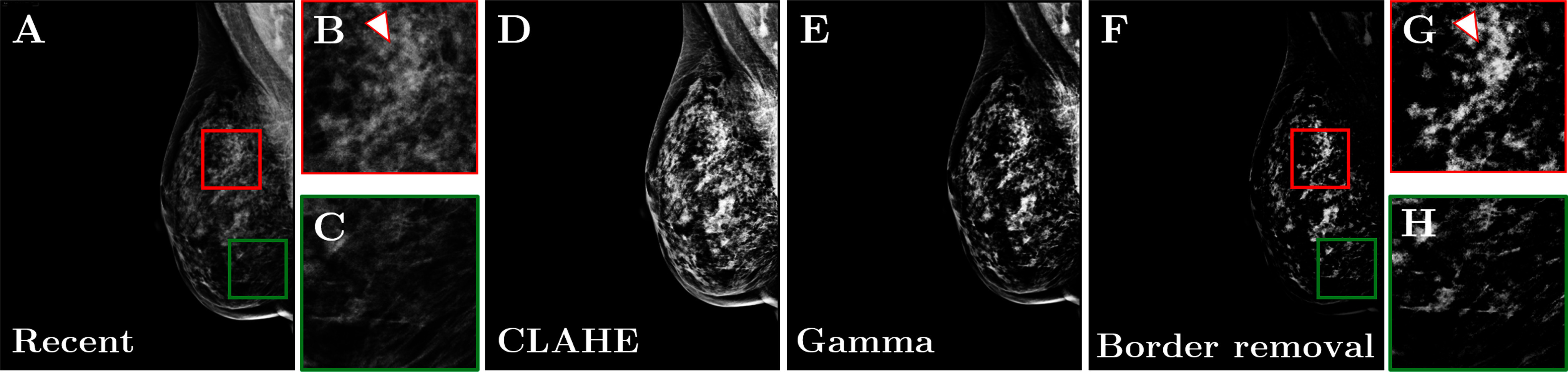
Effect of the pre-processing (BI-RADS breast density category *d*). **(A)** Original most recent mammographic view. **(B)** Zoomed region marked by the red square in **A**, showing an area with a malignant mass (indicated by the arrow). **(C)** Zoomed region marked by the green square in **A**, showing an area without masses. **(D)** Image after CLAHE. **(E)** Image after gamma correction. **(F)** Final pre-processed image after border removal. **(G)** Zoomed region marked by the red square in **F**, showing the same area as **B**, after pre-processing. **(H)** Zoomed region marked by the red square in **F**, showing the same area as **C**, after pre-processing. *CLAHE:* Contrast Limited Adaptive Histogram Equalization.

**Figure 4.**
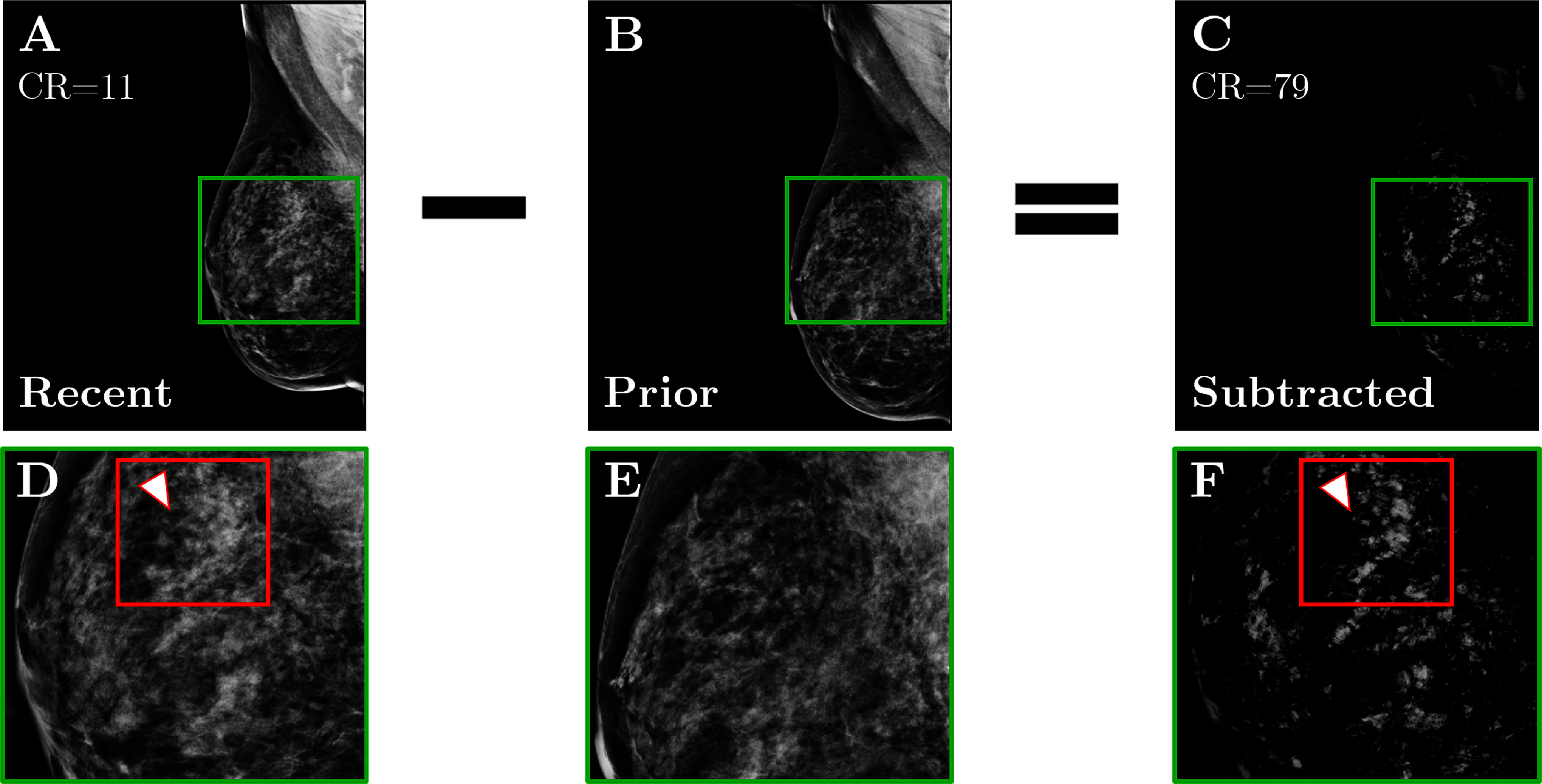
Example of temporal subtraction (BI-RADS breast density class *d*). **(A)** Most recent mammographic view. **(B)** Prior mammographic view. **(C)** The result of subtracting the registered version of **B** from **A**, where the Contrast Ratio (CR) has increased 7 times after subtraction. **(D-F)** Zoomed regions marked by the green squares in **A-C**, where the red squares enclose a new malignant mass that was not subtracted.

Pre-processing was applied using Contrast Limited Adaptive Histogram Equalization (CLAHE), gamma correction, and border removal to enhance image contrast and suppress background noise (Fig. 3). Subsequently, image registration was performed using the Demons algorithm to compensate for variations in breast positioning, compression, and shape between sequential screenings. Temporal subtraction was then applied to subtract the prior registered image from the recent one (Fig. 4). The application of pre-processing, image registration, and temporal subtraction resulted in a notable 69% reduction in image background intensity, highlighting the effective removal of overlapping regions and areas that remained unchanged between the screenings. The subtracted images exhibited an average contrast ratio 4 times higher than that of the most recent mammographic views with the same pre-processing (Supplemental Material; Fig. 3).

Breast masses segmentation was performed through a three-step process involving thresholding, morphological operations, and removal of peripheral pixels. This approach effectively isolated high-intensity regions corresponding to potential masses while eliminating background noise, artifacts, and irrelevant peripheral areas. The resulting segmented Regions Of Interest (ROIs) were subsequently used for feature extraction and classification into true masses or normal tissue. It is important to emphasize that none of the masses (BI-RADS benign or malignant) were removed during these steps (Fig. 4). The computational time required was 15 minutes per image pair, on a Dell Latitude 5430 equipped with a 12th Gen Intel Core™i7-1255 U 1.7 GHz processor (Dell Inc., Round Rock, TX, USA).

### B. Feature Extraction and Selection for Classification

During the feature selection process, utilizing a majority-rule-based combination of all ranking methods assured effective identification of the features with the most important contribution to each classification round. In total, 19 features were selected for the first classification round (Supplemental Material; Table I) including: 14 GLCM features (Correlation 90$^{\circ }$ D2, Correlation Mean D1, Correlation Mean D2, Correlation 90$^{\circ }$ D3, Correlation 45$^{\circ }$ D2, Correlation 0$^{\circ }$ D2, Correlation 135$^{\circ }$ D1, Correlation Mean D3, Correlation 45$^{\circ }$ D1, Correlation 90$^{\circ }$ D3, Correlation 0$^{\circ }$ D2, Correlation 0$^{\circ }$ D3, Correlation 135$^{\circ }$ D2, Correlation 90$^{\circ }$ D1), 2 intensity-based features (Maximum intensity, Mean intensity), 2 shape-based features (Solidity, Extent) and 1 FOS feature (STD). In the second classification round, the 17 selected features (Supplemental Material; Table II) included 10 shape-based features (Major Axis Length, Perimeter, Area, Minor Axis Length, Equivalent Diameter, Extent, Convex Area, Filled Area Circularity, Solidity) and 7 GLCM features (Correlation 135$^{\circ }$ D3, Correlation 0$^{\circ }$ D3, Correlation 45$^{\circ }$ D3, Correlation Mean D3, Correlation 0$^{\circ }$ D2, Correlation 135 D2, Correlation STD D2).

### C. Training and Comparison of Classifier Models

The results of the first classification round, i.e., detected ROIs classified as normal tissue vs. true masses, using Leave-One-Patient-Out (LOPO) cross-validation (CV), are presented in Table 2. The performance of the various approaches ranged between 76.7 - 86.5% sensitivity, 92.8 - 99.4% specificity, 92.7 - 99.2% accuracy, and 0.88 - 0.92 Area Under the receiver operating characteristics Curve (AUC). The classifiers were first compared based on their sensitivity, which is crucial for correctly identifying breast masses. Other metrics were also considered (i.e., accuracy, specificity, and AUC) for a more complete evaluation of the classifier's performance. Statistical analysis was conducted to determine the significance of the observed performance differences among the various classifiers. Using the extended McNemar test [16], the performance of ensemble voting emerged as statistically significant when compared to all classifiers (p $< $ 0.001). Ensemble voting achieved the highest and most stable performance, with a sensitivity of 86.5% (95% CI: 81.7% - 91.4%), specificity of 97.2% (95% CI: 96.9% - 97.5%), accuracy of 97.1% (95% CI: 96.8% - 97.3%), and an AUC of 0.92 (95% CI: 0.89 - 0.94%). Ensemble voting combined k-NN, SVM, MLP, ADA, and GB, in a soft voting scheme. LDA was implemented with a linear discriminant criterion and without prior probabilities. For k-NN, k was set to 11 with a nearest tie-breaking algorithm. An rbf kernel was selected for the SVM. The parameters of each classifier were selected iteratively to optimize the classification accuracy. The ANN architecture selected consisted of five hidden layers. Activation functions included a Rectified Linear Unit (ReLU) in the input and hidden layers, while the output layer employed a softmax function. An Adam optimizer was used, with a batch size set to 128, a learning rate of 0.0001, and the network was trained for 100 epochs. k-fold CV performed (Supplemental Material; Fig. 4), utilizing k = 5 and 10, which verified the robustness of this method. The number of k was selected based on the number of patients in this classification round, which was 100, in order to create an even number of folds per patient, and 2 different k-fold sizes.

**TABLE 2:** Comparison of the Classification Results of the Detected Regions as Normal Tissue or Masses, Using Leave-One-Patient-Out Cross-Validation

**Classifier**	**Sensitivity** **[%]**	**Specificity** **[%]**	**Accuracy** **[%]**	**AUC**
**LDA**	81.35	94.18	94.03	0.88
**k-NN**	82.90	95.90	95.74	0.89
**SVM**	86.53	96.22	96.10	0.91
**RF**	76.68	98.72	98.45	0.88
**MLP**	86.01	97.60	97.46	0.92
**ADA**	86.53	92.80	92.72	0.9
**BAG**	76.68	98.48	98.22	0.88
**GB**	86.53	94.82	94.72	0.91
**Voting**	**86.53**	**97.19**	**97.06**	**0.92**
**ANN**	84.97	99.41	99.23	0.92

Various classifiers were employed for the classification of masses as BI-RADS benign or biopsy-confirmed malignant using LOPO CV. The results are summarized in Table 3. The sensitivity, specificity, accuracy, and AUC ranged between 73.8 - 94.2%, 72.2 - 95.6%, 77.7 - 94.8%, and 0.77 - 0.95, respectively. The most successful classification scheme was based on an ANN, achieving a sensitivity of 94.2% (95% CI: 89.7% - 98.7%), specificity of 95.6% (95% CI: 91.3% - 99.8%), accuracy of 94.8% (95% CI: 91.7% - 97.9%), and an AUC of 0.95 (95% CI: 0.92 - 0.98). The improvement in performance was statistically significant (p $< $ 0.001). In addition, the Matthews Correlation Coefficient (MCC = 0.896) and Cohen's Kappa ($\kappa$ = 0.865) were calculated [17]. These high values indicate that the classifier predictions are highly consistent with the true labels, reflecting strong correlation and almost perfect agreement, and demonstrating robust performance even in the presence of class imbalance. The ANN architecture was the same as before, but only 1 hidden layer was used. In this case, the features were not pre-processed to avoid overfitting. This superior performance is attributed to its optimized architecture, which effectively captures complex nonlinear relationships in the high-dimensional feature space, enabling improved class separability and more reliable distinction between BI-RADS benign and biopsy-confirmed malignant masses. LDA was implemented with a linear discriminant criterion and without prior probabilities. For the k-NN, k was set to 5 with a nearest tie-breaking algorithm. The rbf kernel was used for the SVM. Ensemble voting combined LDA, RF, and GB in a soft voting scheme. The classification performance was also assessed through k-fold CV, employing both 5 and 13 folds. The number of k was selected based on the number of patients with masses (BI-RADS benign and biopsy-confirmed malignant) in this classification round, which was 65, in order to create an even number of folds per patient, and 2 different k-fold approaches. The algorithm demonstrated consistent and robust performance for both (Supplemental Material; Fig. 5).

**TABLE 3:** Comparison of the Classification Results of the Suspicious Masses as BI-RADS Benign or Biopsy-Confirmed Malignant, Using Leave-One-Patient-Out Cross-Validation

**Classifier**	**Sensitivity** **[%]**	**Specificity** **[%]**	**Accuracy** **[%]**	**AUC**
**LDA**	80.58	87.78	83.94	0.84
**k-NN**	73.79	82.22	77.72	0.78
**SVM**	74.76	84.44	79.27	0.8
**RF**	82.52	80.00	81.35	0.81
**MLP**	77.67	85.56	81.35	0.82
**ADA**	82.52	72.22	77.72	0.77
**BAG**	78.64	80.00	79.27	0.79
**GB**	84.47	81.11	82.90	0.83
**Voting**	81.55	87.78	84.46	0.85
**ANN**	**94.17**	**95.56**	**94.82**	**0.95**

## III. Discussion

In this work, an algorithm was developed to automatically detect and classify breast masses, using subtraction of temporally sequential mammographic views. Demons registration outperformed Affine in terms of overall shape alignment and the preservation of subtle changes between screenings. By implementing the proposed algorithm, the contrast ratio was increased 4 times, significantly enhancing the visibility of recent changes in the mammographic images. By reducing the effort and time required for the evaluation, this approach could allow radiologists to efficiently track changes in the mammograms without having to manually refer back to the prior images. Although pre-processing, registration, and temporal subtraction currently take up to 15 minutes per image pair, GPU acceleration and/or multi-threaded CPU implementation are expected to substantially reduce runtime.

Despite some misclassifications, the clinical impact of the proposed approach would have been minimal if applied in practice. While out of 90 benign masses, 4 were wrongly detected as malignant, only 1 patient was falsely burdened with follow-up, as the remaining 3 patients had other malignant masses and they would have been followed up with biopsy, despite the result. Similarly, out of 103 malignant masses, 6 were misclassified as benign, but only 1 patient was falsely cleared, since the masses of the remaining 5 patients were correctly classified as malignant using another view (masses were either classified in CC or MLO view), thus their care would not have been compromised since they would have been biopsied regardless. Although unlikely, a mass that is not changing between screenings could be subtracted and disappear from the final image. However, this would not impose serious clinical consequences since, in such cases, only follow-up is usually recommended.

Robust and effective classification performance hinges on having a balanced dataset. In this study, the implementation of SMOTE played a crucial role in addressing the dataset imbalance, leading to more robust results, particularly in terms of sensitivity. To mitigate potential overfitting, k-fold cross-validation per patient was also employed, to assess performance variation across different training folds. Additionally, hyperparameter tuning in the ANN using dropout regularization, batch normalization, and Gaussian noise helped to reduce reliance on specific neurons and prevent the network from memorizing the training data. In addition, learning curves of loss and accuracy for both training and validation were monitored, and early stopping was applied to further prevent overfitting. A slight performance decrease was observed with k-fold CV, given the reduced training data compared to the 99 patients in LOPO CV. This decline underscores the need for a larger dataset while affirming the algorithm's ability to accurately classify new data. Moving forward, when more data become available, the dataset will be divided into separate training, validation, and test sets to evaluate the model on a completely new patient cohort. This approach will allow for a better understanding of the model's ability to generalize to unseen data.

The subtraction of temporally sequential mammograms for breast malignancy diagnosis is a new approach and, thus, direct comparison with existing studies is challenging. Differences in datasets, pre-processing methodologies, CV techniques, classifiers, and performance evaluation methods further complicate such comparisons. In some studies, the ROIs were randomly divided into training and test sets, leading to the inclusion of regions from the same image, thus the same patient, in both sets [18], [19]. To mitigate such bias, in this study, a CV strategy that was patient-centric, rather than ROI-centric, was adopted. While some algorithms have been proposed for the classification of breast masses as benign or malignant using sequential mammograms and temporal analysis [18], [20], [21], they often fall short of classifying evolving abnormalities. Unlike these studies, the proposed approach focuses on automatically detecting and classifying newly developed or notably altered regions between sequential screenings. Thus, a major contribution of this approach is its ability to capture and classify evolving lesions over time, while, at the same time, maintaining higher classification performance compared to the other studies in the literature (Table 4).

**TABLE 4:** Comparison of Available Studies, in the Literature, for the Classification of Masses as Benign or Malignant Using Sequential Mammograms and Feature-Based Machine Learning

**Reference**	**Nu. of patients**	**Classifier**	**Validation method**	**AUC**
Hadjiiski et al. (2001) [20]	140	LDA	leave-one-out (per patient)	0.88
Timp et al. (2007) [12]	465	SVM	20-fold CV (per ???$^{1}$)	0.77
Bozek et al. (2014) [18]	60	LDA	leave-one-out (per ???$^{1}$)	0.90
**Proposed**	**100**	**ANN**	**leave-one-out CV** **(per patient)**	**0.95**

^0^No information was provided on how the data were divided into training and test sets (per ROI, per image, or per patient).

Some limitations of this study should be noted. First, the dataset is relatively small, comprising 100 patients (400 images), which may limit the generalizability of the findings across different populations and imaging systems [22]. The inclusion criteria—requiring a normal prior mammogram and a biopsy-confirmed mass in the recent mammogram—further restrict diversity and contribute to data imbalance. Additionally, the need for two consecutive annotated mammographic rounds per patient makes data collection complex. Nevertheless, this study serves as a proof-of-concept, demonstrating that subtraction of temporally sequential mammograms can improve classification accuracy. In addition, due to the limited size of the dataset, no external test set was used for the validation of the algorithms. Another key limitation of this study is the lack of external validation using publicly available datasets. Unfortunately, there are no publicly available databases that include (1) two rounds of mammograms per patient, (2) precise, detailed annotations for benign and malignant abnormalities, or (3) biopsy confirmations, and, in some cases, they include scanned, low-resolution images. Another limitation of this study is the aspect of potential differences in annotations when more experts evaluate the same mammograms, which has not been very rigorously evaluated yet. The computational complexity of the proposed methodology is also a challenge, as pre-processing, registration, and subtraction require significant resources, making real-time clinical deployment difficult. Additionally, training machine learning models is time-consuming, which could further limit scalability in clinical settings.

Future work will focus on expanding the dataset to include a more diverse and representative population, with age-matched groups. This will help address the limitations observed in k-fold CV, where the classification performance slightly dropped due to the algorithm being trained with fewer patients in each fold. Future studies will include separate training, validation, and external test sets to better assess the generalization performance pf the proposed algorithm. When more data are available, new studies will be conducted for the integration of deep learning in order to automate and optimize the image pre-processing and registration pipeline, reducing reliance on manual parameter selection and improving the model's robustness to variations in mammogram quality, patient positioning, and anatomical differences. As larger multi-institutional datasets become available, a more comprehensive error analysis will be conducted to systematically assess the relation of misclassified cases to lesion size, breast density, and other clinical factors. To address computational constraints, future work will focus on optimizing the methodology by leveraging high-performance computing systems, GPU acceleration, and model compression techniques to enhance processing efficiency and ensure feasibility for real-time clinical applications. Although the findings are promising, a prospective clinical trial is required to validate the model's real-world effectiveness, including its impact on diagnostic efficiency and radiologist workload. It is also acknowledged that the performance estimates by breast density subgroups were not stratified; in future studies, algorithmic robustness across breast density categories will be examined to uncover potential biases. The impact of incorporating normal prior mammograms for the prediction of near-term BC occurrence will be investigated. Additionally, the investigation of masses falsely considered normal in the first classification round and those misclassified in the second stage (BI-RADS benign vs. malignant) will take place. Finally, a dedicated robustness analysis to evaluate how sensitive the method is to small misalignments, variations in breast compression, or hardware-related differences between scans across heterogeneous imaging systems remains an important direction for future work.

## IV. Conclusion

In conclusion, the proposed technique demonstrates improved performance using subtraction of temporally sequential mammograms for breast mass detection and classification, achieving an AUC of 0.95 compared to 0.90 in previous studies. By serving as a reliable “second reader” or “tie breaker,” especially in resource-limited environments, this approach provides a meaningful advancement toward automated CAD systems and has the potential to improve both sensitivity and efficiency in the routine diagnosis of breast abnormalities.

## V. Materials and Methods

A comprehensive description of the materials and methods is available in the *Supplementary Materials*.

## Supplementary Materials

The Supplementary Materials contain a comprehensive description of the study methodology.

Supplementary Materials

## CONFLICT OF INTEREST

The authors declare that they have no conflict of interest.
