# Supplementary material for: Subtraction of Temporally Sequential Digital Mammograms: Enhancing the Detection and Classification of Malignant Masses in Breast Imaging
Source: IEEE Open J Eng Med Biol. 2025 Oct 23;6:591–7. doi: 10.1109/OJEMB.2025.3624977 (PMC12599895; doi:10.1109/OJEMB.2025.3624977)
Supplement: Supplementary Materials [file supp1-3624977.pdf]

Supplementary Materials

Subtraction of Temporally Sequential Digital Mammograms: Enhancing the Detection and Classification of Malignant Masses in Breast Imaging

Kosmia Loizidou, *Member, IEEE*, Galateia Skouroumouni, Gabriella Savvidou, Anastasia Constantinidou, Eleni Orphanidou Vlachou, Anneza Yiallourou, Costas Pitrism *Member, IEEE*, and Christos Nikolaou

I. Materials and Methods

A. Study Design and Data Collection

IN this retrospective study, 100 pairs of full-field digital mammograms were obtained from screenings conducted between 2016 and 2023, from different local healthcare facilities, including the Cyprus Population Screening Program in Aglantzia and Linopetra, Limassol, and Nicosia General Hospitals. The population included women 40 to 81 years of age (mean ± standard deviation, 60.2 ± 9.6), with normal or benign (BI-RADS 1 & 2) mammograms in their first screening round. Their most recent mammograms contained either no masses (BI-RADS 1), benign masses (BI-RADS 2), or biopsy-confirmed malignant masses, with an average interval of 2.4 years between screenings. The normal population consisted of the BI-RADS 1 and BI-RADS 2 participants, while the participants associated with malignancies formed the malignant population. Ethical considerations were addressed, and the study received approval from the Institutional Review Board (Cyprus National Bioethics Committee). Informed consent forms were collected during the most recent screening round. This study was conducted in accordance with the ethical principles of the Declaration of Helsinki.

Two mammographic views of the breast (Cranio-Caudal (CC) and Medio-Lateral Oblique (MLO)) from two sequential screening rounds were collected from each participant, resulting in a total of 400 images. Two clinicians (C.N., radiologist with 27 years of experience, and A.Y., consultant breast surgeon with 10 years of experience) identified the eligible patients. Two radiologists (G.Sk. with 6 years of experience and E.O.V. with 5 years of experience), outlined the border of each mass for both BI-RADS benign and suspicious masses. Inter-observer agreement between the two radiologists was high, with an observed agreement of 97.41% and a Cohen's kappa of 0.95, indicating almost perfect reliability of the ground truth labels [1]. Discrepancies were resolved by consensus. Subsequently, suspicious cases were biopsied, followed by histopathological analysis, confirming their malignant nature. The biopsy confirmations were performed by each healthcare provider's pathology department, as per standard of care. The information was collected by a clinician and a researcher (A.C., academic medical oncologist with over 10 years of experience, and G.Sa., postgraduate researcher at the medical school with 5 years of experience).

Fifty percent of the population had none or benign findings in the first round of screening, and none or benign findings in the most recent mammogram (35 with no visible masses and 15 with only BI-RADS benign masses). Cases in which benign findings emerged in the most recent screening with prior normal mammograms were included, along with cases in which benign findings appear both in prior and recent views, in order to investigate whether and how these findings evolved over time, ensuring effective analysis. The remaining 50% of the patients had normal or benign priors but at least one new biopsy-confirmed malignant mass in the most recent mammographic views. All prior mammograms had been reported as normal, with no traces of suspicious lesions, at the time of screening by the associated radiologists. This dataset included sequential mammographic images, precise annotations of each individual mass, which were used as a reference, and biopsy confirmations for the cases associated with suspicious masses. In total, 193 masses appeared, 90 BI-RADS benign (BI-RADS 2) and 103 biopsy-confirmed malignant. The dimensions of the mammographic images were 4096 x 3328 pixels in 8-bit DICOM format. This dataset is currently open-access (https://zenodo.org/records/11446259).

B. Breast Mass Segmentation and Detection

This study extends our previously published work [2], which focused on the detection and BI-RADS classification of microcalcifications. In this study, breast masses –a different type of breast abnormality –were taken into consideration, and biopsy confirmations were added for the suspicious cases. The algorithm was enhanced, and various methodologies were extended and optimized for better performance. Fig. 1 demonstrates the proposed methodology for the diagnosis of BI-RADS benign and biopsy-confirmed malignant breast masses. To prepare the mammograms for the subsequent analysis, a series of preprocessing steps were implemented, including: normalization and the application of three sequential filtering techniques (Contrast Limited Adaptive Histogram Equalization (CLAHE) [3], gamma correction [4], and border removal [5]).

Accurate registration is necessary to compensate for multiple factors affecting breast imaging, including variations in breast compression, alterations in breast shape, discrepancies in the presence of the pectoral muscle in the MLO view, and potential human errors occurring at the time of screening [6]. Demons registration [7], a nonrigid image alignment technique based on local deformations, was selected over other local and global registration techniques [8], due to its superior performance in terms of alignment accuracy. Specifically, the Demons and Affine registration techniques were evaluated and compared based on the residuals of the registration and subtraction process, which represent the sum of the remaining pixels after subtraction (Fig. 2). Demons demonstrated better overall shape

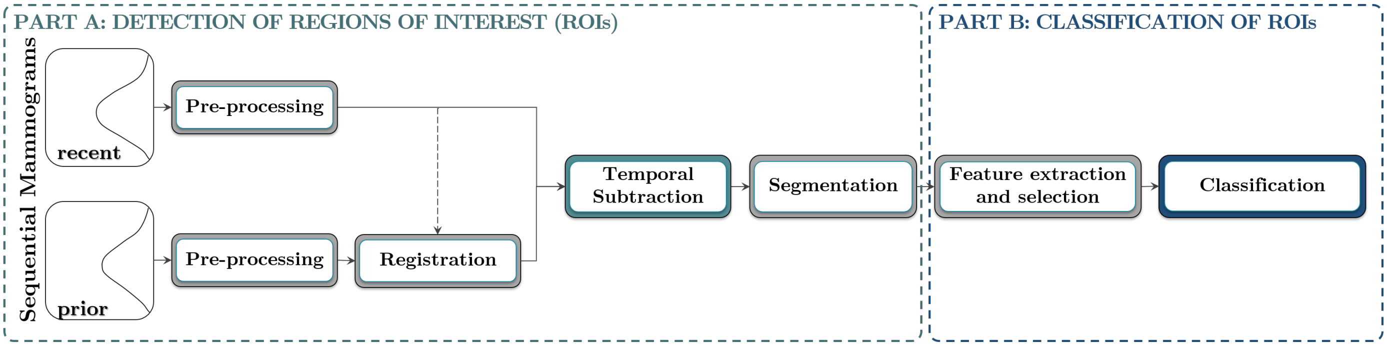

Fig. 1. Diagram of the proposed methodology.

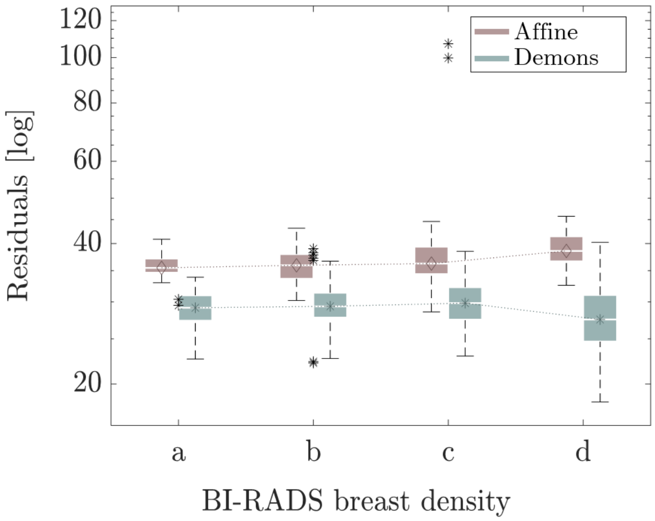

Fig. 2. Plot comparing Affine and Demons registration based on the residuals of the subtraction, in logarithmic scale, for the four categories of breast density as defined by the BI-RADS. *BI-RADS:* Breast Imaging Reporting and Data System

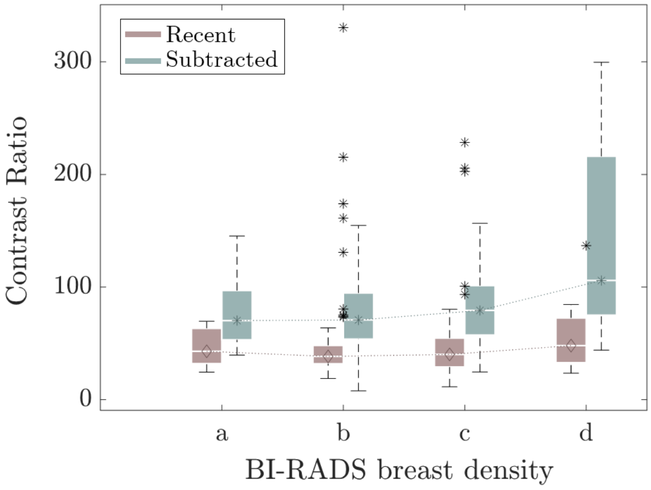

Fig. 3. Plot comparing the contrast ratio, in logarithmic scale, of the processed recent image and the image created by temporal subtraction, for the four categories of BI-RADS breast density. The contrast ratio increased in all cases, indicating that temporal subtraction was successful for all breast densities. *BI-RADS:* Breast Imaging Reporting and Data System

alignment, with fewer residuals, while preserving the subtle changes between sequential mammograms. Therefore, Demons was selected as the most suitable method for this study, as it effectively accounted for the local and non-linear shape deformations of the breast. The two-stage registration process involved overall shape matching, followed by detailed, local registration. The prior mammographic view was registered to the most recent one, and then subtracted from the latter, effectively eliminating the overlapping regions that have remained unchanged between the screenings. The masses in the recent mammograms remained unaffected by the registration process, since it was the prior mammograms that were modified to align with the corresponding recent ones. The contrast ratio of the resulting subtracted image, calculated as the ratio of maximum to the average intensity, was compared to that of the recent mammographic view after pre-processing, to assess the effectiveness of temporal subtraction (Fig. 3). The subtracted image was further processed using unsharp mask filtering, a spatial filter that enhances a range of high frequencies [9].

The segmentation of masses was carried out in three steps: (1) thresholding, (2) application of morphological operations, and (3) removal of periphery pixels. The process started with thresholding, which converted the image into a binary format, thereby eliminating low-intensity normal areas. The threshold value was determined through histogram analysis and optimization of the global classification rate during training. After the thresholding, morphological operations were applied to refine the binary image. Erosion was first used to eliminate isolated pixels that may have resulted from misalignment during registration or from thresholding artifacts. This operation was performed with a 2-pixel radius, smaller than the average size of masses, ensuring that adjacent regions were not unintentionally merged. Next, closing was applied using a 10-pixel radius to merge neighboring pixels and identify the constituents of each mass. This radius size was chosen after a comparison with the ground truth. In the final step, high-intensity regions at the periphery of the breast, which likely corresponded to the skin, were removed, as masses are not expected to form in that area. The border of the entire breast region was identified, allowing for the removal of all high-intensity regions along the border. The remaining regions were

TABLE I. Ranking of the features for the first classification round (normal vs. actual mass), using different feature selection techniques. The final selection, using the majority rule, is in bold.

t-test	MRMR	FI-ET	FI-RF	FI-XGB	SelectKBest	SFS	SBS
**Corr.[1] 90 D3**	**STD**	Variance	Variance	**Corr. 90 D1**	**Mean Intensity**	Orientation	Area
**Corr. 90 D2**	**Corr. Mean D1**	**STD**	**STD**	**Corr. Mean D2**	**STD**	EN[6]	ED
**Corr. 0 D3**	**Corr. Mean D3**	Smoothness	Smoothness	**STD**	Variance	ED	Perimeter
**Corr. 45 D2**	Corr. STD D1	**Solidity**	**Solidity**	**Corr. Mean D3**	Entropy	**Solidity**	Skewness
**Corr. 135 D2**	**Corr. 0 D3**	**Mean Intensity**	**Mean Intensity**	**Corr. Mean D1**	**Corr. 45 D1**	**Extent**	Con. 90 D1
**Corr. 0 D2**	Kurtosis	**Corr. Mean D3**	**Extent**	Solidity	**Corr. 90 D1**	**Max Intensity**	Con. Mean D1
**Corr. Mean D1**	**Corr. 90 D2**	**Corr. 0 D2**	**Corr. Mean D2**	Con. Mean D2	**Corr. 135 D1**	Skewness	Energy 135 D1
Con.[2] Mean D3	**Max Intensity**	**Corr. Mean D2**	**Corr. 0 D2**	Area	**Corr. Mean D1**	**STD**	Con. STD D2
Con. 90 D3	**Corr. 45 D2**	**Extent**	**Corr. Mean D3**	Energy Mean D2	Energy 0 D1	Entropy	**Corr. 45 D2**
Con. 45 D3	**Corr. 135 D2**	**Corr. Mean D1**	**Corr. 90 D2**	Circularity	Energy Mean D1	Con. Mean D1	**Corr. Mean D2**
Con. 135 D3	Energy 135 D3	Energy 135 D1	**Max Intensity**	Homo.[5] Mean D1	Con. 45 D2	Corr. 0 D1	Homo. 135 D2
**Corr. 45 D1**	**Corr. 45 D1**	**Max Intensity**	Corr. 0 D1	Con. 45 D1	Con. 135 D2	**Corr. 45 D1**	Con. 90 D3
Corr. 45 D3	**Corr. 90 D3**	**Corr. 45 D2**	**Corr. Mean D1**	Con. STD D1	Con. Mean D2	**Corr. 135 D1**	Con. 135 D3
Corr. 135 D3	**Corr. 0 D2**	**Corr. 90 D2**	MiAL[3]	**Extent**	**Corr. 0 D2**	Energy 90 D1	**Corr. 90 D3**
**Corr. 135 D1**	Entropy	**Corr. 90 D3**	Shape Ratio	MiAL	**Corr. 45 D2**	Corr. 0 D3	Homo. 0 D3
Con. 0 D3	**Corr. 135 D1**	**Corr. 135 D2**	**Corr. 90 D1**	Con. 135 D2	**Corr. 90 D2**	**Corr. Mean D3**	Circularity
Con. 45 D2	Corr. 135 D3	**Corr. 45 D1**	**Corr. 90 D3**	Con. Mean D1	**Corr. 135 D2**	**Energy 0 D3**	Shape Ratio
Con. 135 D2	**Corr. Mean D2**	Energy Mean D2	**Corr. 0 D3**	Perimeter	**Corr. Mean D2**		Breast Density
Con. Mean D2	**Mean Intensity**	Corr. 0 D1	ED[4]	Energy 135 D2	Con. 0 D3		
**Corr. 90 D1**	**Solidity**	Area	**Corr. 135 D1**	**Corr. 90 D2**	Con. 45 D3		

[1]Corr.: Correlation [2]Con.: Contrast [3]MiAL: Minor Axis Length [4]ED: Equivalent Diameter [5]Homo.: Homogeneity [6]Euler Number

TABLE II. Ranking of the features for the second classification round (BI-RADS benign vs. biopsy-confirmed malignant masses), using different feature selection techniques. The final selection, using the majority rule, is in bold.

t-test	MRMR	FI-ET	FI-RF	SelectKBest	SFS	SBS
**Area**	**Area**	**Area**	**Area**	**Area**	**MaAL**	**Area**
**MaAL[1]**	**MaAL**	**MaAL**	**MaAL**	**MaAL**	Orientation	**MaAL**
**MiAL[2]**	**MiAL**	**MiAL**	**MiAL**	**MiAL**	**Solidity**	**MiAL**
**Convex Area**	**Convex Area**	**Convex Area**	**Convex Area**	**Convex Area**	**Extent**	**Filled Area**
**Filled Area**	Euler Number	**Filled Area**	**Filled Area**	**Filled Area**	**Perimeter**	**ED**
**ED[3]**	**ED**	**ED**	**ED**	**ED**	Mean Intensity	**Perimeter**
**Solidity**	**Extent**	**Solidity**	**Extent**	**Solidity**	Min Intensity	Max Intensity
**Extent**	**Perimeter**	**Extent**	**Perimeter**	**Extent**	Max Intensity	Con. 0 D1
**Perimeter**	Max Intensity	**Perimeter**	Correlation 45 D1	**Perimeter**	STD	Con. 45 D1
**Corr.[4] 0 D2**	Corr. 0 D1	**Corr. 0 D2**	Energy STD D1	**Corr. 0 D2**	Variance	Con. 90 D1
Corr. 45 D2	**Corr. STD D2**	Corr. 45 D2	**Corr. 0 D2**	Corr. 45 D2	Con.[6] 0 D1	Con. Mean D1
**Corr. 135 D2**	Energy STD D2	**Corr. 135 D2**	**Corr. 135 D2**	**Corr. 135 D2**	Con. 45 D1	Corr. 45 D1
Corr. Mean D2	Homo.[5] 45 D2	Corr. Mean D2	**Corr. STD D2**	Corr. Mean D2	Con. 90 D2	Energy 90 D1
**Corr. STD D2**	**Corr. 0 D3**	Homo. STD D2	**Corr. 0 D3**	**Corr. STD D2**	Breast Density	Con. 0 D2
**Corr. 0 D3**	**Corr. 45 D3**	**Corr. 0 D3**	**Corr. 45 D3**	**Corr. 0 D3**	Age	Energy Mean D2
**Corr. 45 D3**	**Corr. 135 D3**	**Corr. 45 D3**	**Corr. 135 D3**	**Corr. 45 D3**		Energy 45 D3
Corr. 90 D3	**Corr. Mean D3**	**Corr. 135 D3**	**Corr. Mean D3**	Corr. 90 D3		**Corr. 135 D3**
**Corr. 135 D3**	Circularity	**Corr. Mean D3**	**Circularity**	**Corr. 135 D3**		Shape Ratio
**Corr. Mean D3**	Compactness	**Circularity**	Compactness	**Corr. Mean D3**		
**Circularity**	Age	**Shape Ratio**	Shape Ratio	**Circularity**		

[1]MaAL: Major Axis Length [2]MiAL.: Minor Axis Length [3]ED: Equivalent Diameter [4]Corr.: Correlation [5]Homo: Homogeneity [6]Con: Contrast

then considered as potential masses or ROIs.

C. Feature Extraction and Selection for Classification

Numerous features were extracted from every segmented ROI to first separate the actual masses from the falsely segmented normal tissue and then classify the masses as benign or malignant using various classifiers. In total, 98 features were extracted, including 2 epidemiological and 96 image characteristics (shape-based, intensity-based, first-order statistics (FOS), and gray level co-occurrence matrix (GLCM) features) [10], [11]. Feature extraction was performed using MATLAB 2024b. For each segmented ROI, a feature vector consisting of 98 values was generated. The epidemiological features considered included the age at the time of the most recent mammograms and BI-RADS breast density. Regarding the GLCM features, each was derived at 0, 45, 90, and 135 degrees, along with the mean and standard deviation (STD), i.e., 24 values for each different offset D ($D_1 = 5$, $D_2 = 15$, and $D_3 = 25$ pixels).

However, not all of these features contributed to the classification and diagnosis and, therefore, had to be removed from the process. To identify the most relevant features, a

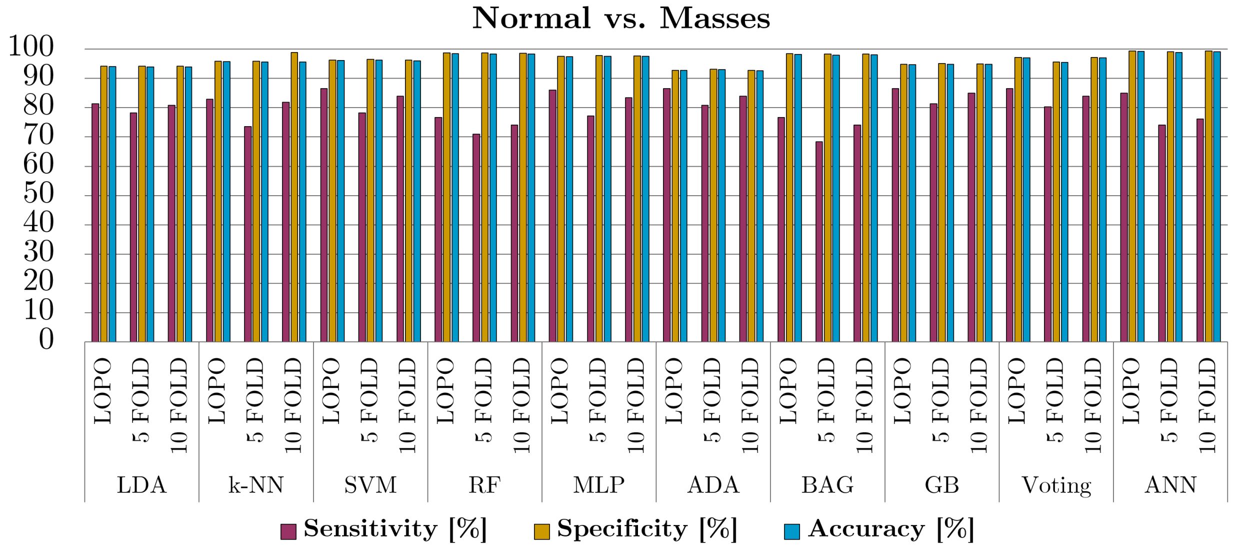

Fig. 4. Classification results of the detected regions as normal tissue or masses, using different classifiers and cross-validation methods. *LDA*: Linear Discriminant Analysis; *k-NN*: k-Nearest Neighbors; *SVM*: Support Vector Machine; *RF*: Random Forest; *MLP*: Multi-Layer Perceptron; *ADA*: Adaboost; *BAG*: Bagging; *GB*: Gradient Boosting; *ANN*: Artificial Neural Network; *LOPO*: Leave-One-Patient-Out

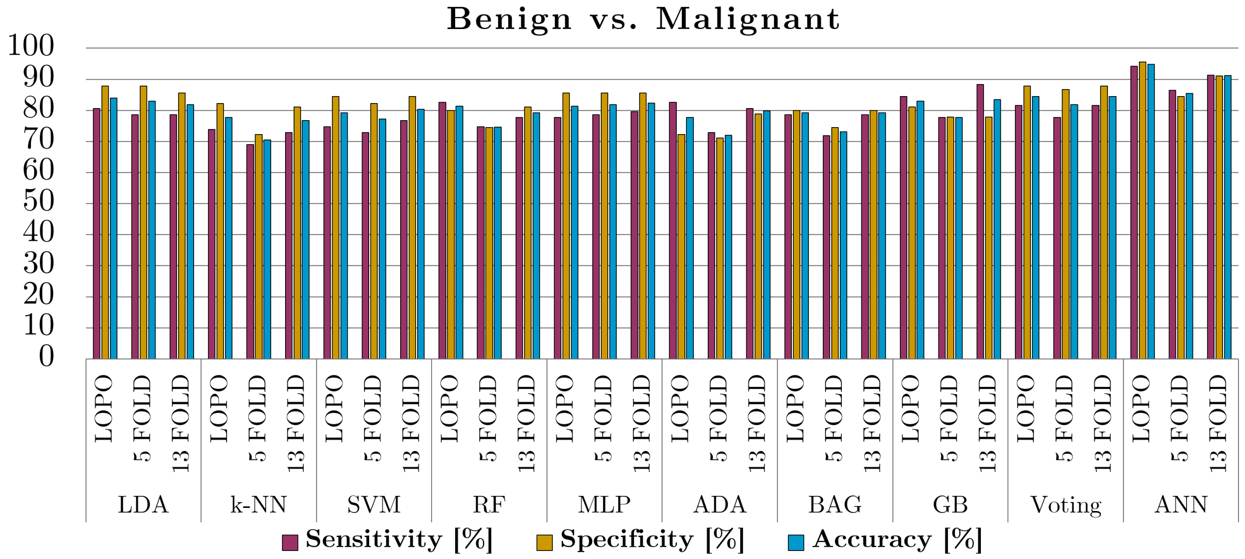

Fig. 5. Classification results of the masses as BI-RADS benign or biopsy-confirmed malignant, using different classifiers and cross-validation methods. *LDA*: Linear Discriminant Analysis; *k-NN*: k-Nearest Neighbors; *SVM*: Support Vector Machine; *RF*: Random Forest; *MLP*: Multi-Layer Perceptron; *ADA*: Adaboost; *BAG*: Bagging; *GB*: Gradient Boosting; *ANN*: Artificial Neural Network; *LOPO*: Leave-One-Patient-Out

combination of eight feature selection methods was employed, which included: (1) t-test, (2) Maximum Relevance-Minimum Redundancy (MRMR), (3) Feature Importance using Extra Trees (FI-ET), (4) Feature Importance using Random Forest (FI-RF), (5) Feature Importance using XGBoost (FI-XGB), (6) SelectKBest, (7) Sequential Forward Selection (SFS), and (8) Sequential Backward Selection (SBS) [12]–[14]. This ensemble approach reduces the dependency on any single ranking criterion, thereby mitigating potential bias and improving the reliability of classification compared to individual methods. Scores were generated for each feature using methods (1) to (6), and the top 20 features with the highest scores were selected to streamline the procedure and avoid unnecessary complexity. To further optimize the feature set, SFS and SBS were used to assess classification performance using different subsets of the selected features, again optimized to identify the 20 features with the highest performance. A majority-rule approach was applied to consolidate the results across all methods, and features common to at least three techniques were retained for the final feature set (Tables I & II). This approach was selected to ensure that only the most significant features were included, and the potential for bias in the selection process was minimized by relying on multiple independent feature selection methods. All feature selection techniques were

applied only to the training set, to avoid bias.

D. Training and Comparison of Classifier Models

For the classification, nine classifiers were evaluated including: Linear Discriminant Analysis (LDA) [15], k-Nearest Neighbors (k-NN) [16], Support Vector Machine (SVM) [17], Random Forest (RF) [18], Multi-Layer Perceptron (MLP) [19], Adaboost (ADA) [20], Bagging (BAG) [21], Gradient Boosting (GB) [22], and ensemble voting [20]. In the case of k-NN, various nearest neighbor values (1, 3, 5, 7, 9, and 11) were tested to identify the optimal configuration. For SVM, various kernel functions were evaluated, such as linear, polynomial, and Radial Basis Function (rbf). The ensemble voting method was subject to evaluation in both hard and soft schemes. Furthermore, several artificial neural network (ANN) [16] architectures were tested, with network parameters fine-tuned based on validation loss and accuracy. The tuning process involved adjusting key hyperparameters, including batch size, learning rate, number of hidden layers, number of neurons per layer, activation functions, and optimizers. The optimal configuration was determined by systematically evaluating different parameter combinations to achieve the best classification performance during training. In total, more than 20 classifiers were initially evaluated for the classification. The classifiers demonstrating the most robust performance were selected for detailed analysis. These included a mix of linear, nonlinear, and ensemble methods to provide a comprehensive benchmark across different learning paradigms.

The classifiers were trained and tested using two validation approaches: LOPO and k-fold-patient Cross-Validation (CV). In both cases, validation per patient is crucial and necessary to avoid bias from the inclusion of images from the same patient in both the training and test sets and artificially improve the algorithm's accuracy. In both LOPO and k-fold CV, each patient's data were exclusively assigned to either the training or test set, eliminating any overlap between the two. Additionally, each mass was treated independently, as the MLO and CC views capture it from different angles, potentially leading to distinct feature representations and classifications. This approach is equivalent to radiological evaluation, where, in some cases, even the radiologists cannot identify the mass in both views. In two consecutive classification rounds, the falsely detected regions were first eliminated, and then the masses were classified as BI-RADS benign or biopsy-confirmed malignant. To address class imbalance, the synthetic minority oversampling technique (SMOTE) was employed [23]. SMOTE works by generating synthetic instances of the minority class by interpolating between existing minority samples in the feature space. When training the model, SMOTE generated synthetic samples of the minority class, whether benign or malignant, based on the training data available for each fold. This ensured that the classifier was not biased toward the majority class. The process was repeated each time during CV, adjusting the balance based on the data of the patient left out in each iteration. This technique allowed for a more robust model capable of handling the class imbalance while training on a balanced dataset at every step.

The classification performance was assessed by computing key evaluation metrics, including sensitivity, specificity, accuracy, and the AUC. Optimal cutoff values were chosen to minimize FP and False Negative (FN) instances.

References

[1] M. L. McHugh, "Interrater reliability: the kappa statistic," *Biochemia medica*, vol. 22, no. 3, pp. 276–282, 2012.
[2] K. Loizidou, G. Skouroumouni, C. Pitris, and C. Nikolaou, "Digital subtraction of temporally sequential mammograms for improved detection and classification of microcalcifications," *European radiology experimental*, vol. 5, no. 1, pp. 1–12, 2021.
[3] S. Agrawal, R. Rangnekar, D. Gala, S. Paul, and D. Kalbande, "Detection of breast cancer from mammograms using a hybrid approach of deep learning and linear classification," in *2018 International Conference on Smart City and Emerging Technology (ICSCET)*, pp. 1–6, IEEE, 2018.
[4] S.-C. Huang *et al.*, "Efficient contrast enhancement using adaptive gamma correction with weighting distribution," *IEEE Transactions on Image Processing*, vol. 22, no. 3, pp. 1032–1041, 2013.
[5] R. Gonzalez *et al.*, *Digital image processing using MATLAB*, pp. 80–193, 486–534. Gatesmark Publishing, 2nd ed., 2010.
[6] K. Marias, C. Behrenbruch, S. Parbhoo, A. Seifalian, and M. Brady, "A registration framework for the comparison of mammogram sequences," *IEEE Transactions on Medical Imaging*, vol. 24, no. 6, pp. 782–790, 2005.
[7] X. Pennec, P. Cachier, and N. Ayache, "Understanding the "demon's algorithm": 3d non-rigid registration by gradient descent," in *International Conference on Medical Image Computing and Computer-Assisted Intervention*, pp. 597–605, Springer, 1999.
[8] Y. Díez, A. Oliver, X. Lladó, J. Freixenet, J. Martí, J. Vilanova, and R. Martí, "Revisiting intensity-based image registration applied to mammography," *IEEE Transactions on Information Technology in Biomedicine*, vol. 15, no. 5, pp. 716–725, 2011.
[9] H. P. Chan, C. J. Vyborny, H. MacMahon, C. E. Metz, K. Doi, and E. A. Sickles, "Digital mammography. roc studies of the effects of pixel size and unsharp-mask filtering on the detection of subtle microcalcifications.," *Investigative Radiology*, vol. 22, no. 7, pp. 581–589, 1987.
[10] K. Loizidou, G. Skouroumouni, G. Savvidou, A. Constantinidou, C. Nikolaou, and C. Pitris, "Identification and classification of benign and malignant masses based on subtraction of temporally sequential digital mammograms," in *2022 44th Annual International Conference of the IEEE Engineering in Medicine & Biology Society (EMBC)*, pp. 1667–1670, IEEE, 2022.
[11] K. Loizidou *et al.*, "Computer-aided breast cancer detection and classification in mammography: A comprehensive review," *Computers in Biology and Medicine*, p. 106554, 2023.
[12] P. Diehr, D. C. Martin, T. Koepsell, and A. Cheadle, "Breaking the matches in a paired t-test for community interventions when the number of pairs is small," *Statistics in medicine*, vol. 14, no. 13, pp. 1491–1504, 1995.
[13] C. Ding and H. Peng, "Minimum redundancy feature selection from microarray gene expression data," *Journal of bioinformatics and computational biology*, vol. 3, no. 02, pp. 185–205, 2005.
[14] F. Pedregosa, G. Varoquaux, A. Gramfort, V. Michel, B. Thirion, O. Grisel, M. Blondel, P. Prettenhofer, R. Weiss, V. Dubourg, *et al.*, "Scikit-learn: Machine learning in python," *the Journal of machine Learning research*, vol. 12, pp. 2825–2830, 2011.
[15] S. Mika, G. Ratsch, J. Weston, B. Scholkopf, and K.-R. Mullers, "Fisher discriminant analysis with kernels," in *Neural networks for signal processing IX, 1999. Proceedings of the 1999 IEEE signal processing society workshop.*, pp. 41–48, Ieee, 1999.
[16] N. I. Yassin, S. Omran, E. M. El Houby, and H. Allam, "Machine learning techniques for breast cancer computer aided diagnosis using different image modalities: A systematic review," *Computer methods and programs in biomedicine*, vol. 156, pp. 25–45, 2018.
[17] C. Cortes and V. Vapnik, "Support-vector networks," *Machine learning*, vol. 20, no. 3, pp. 273–297, 1995.
[18] L. Breiman, "Random forests," *Machine learning*, vol. 45, no. 1, pp. 5–32, 2001.
[19] M. R. Mohebian, H. R. Marateb, M. Mansourian, M. A. Mañanas, and F. Mokarian, "A hybrid computer-aided-diagnosis system for prediction of breast cancer recurrence (hpbcr) using optimized ensemble learning," *Computational and structural biotechnology journal*, vol. 15, pp. 75–85, 2017.

[20] E. Bauer and R. Kohavi, "An empirical comparison of voting classification algorithms: Bagging, boosting, and variants," *Machine learning*, vol. 36, no. 1, pp. 105–139, 1999.

[21] L. Breiman, "Bagging predictors," *Machine learning*, vol. 24, no. 2, pp. 123–140, 1996.

[22] J. H. Friedman, "Stochastic gradient boosting," *Computational statistics & data analysis*, vol. 38, no. 4, pp. 367–378, 2002.

[23] N. V. Chawla, K. W. Bowyer, L. O. Hall, and W. P. Kegelmeyer, "Smote: synthetic minority over-sampling technique," *Journal of artificial intelligence research*, vol. 16, pp. 321–357, 2002.
